# Supramolecular Mechanophore Additives Enable Mechanochromic Hydrogels

**DOI:** 10.1002/anie.7699472

**Published:** 2026-09-07

**Authors:** Iulia Scarlat, Jess M. Clough, Christoph Weder

**Affiliations:** ^1^ Adolphe Merkle Institute (AMI) University of Fribourg Fribourg Switzerland; ^2^ NCCR Bio‐inspired Materials University of Fribourg Fribourg Switzerland

**Keywords:** additive, hydrogel, mechanochromic, mechanophore, supramolecular

## Abstract

Mechanochromic hydrogels that report deformation through optical signals require mechanophores that function in aqueous environments, are activated at low force, and allow integration into the hydrogel matrix. Here, we report a new strategy to address these challenges that involves deploying a supramolecular mechanophore as a water‐soluble macromolecular additive. The mechanophore developed for this purpose features a loop‐forming architecture, in which a naphthalene diimide (NDI) quencher and a 9,10‐bis(phenylethynyl)anthracene (BPEA) fluorophore are connected by a short spacer. Intramolecular charge‐transfer interactions between these moieties induce loop formation and quench the BPEA emission. Mechanical deformation unfolds the loop, disrupts the charge‐transfer interaction, and increases the BPEA emission. The functionalization of this loop‐forming motif with poly(oligo(ethylene glycol) methyl ether methacrylate) (pOEGMA) affords a macromolecular water‐soluble additive that can simply be blended into different hydrogel matrices, including cross‐linked poly(acrylamide) and pOEGMA. These hydrogels exhibit a pronounced increase in emission intensity upon swelling, stretching, and compression. Spectroscopic investigations reveal correlations between the applied deformation and the optical response and show that the mechanophore activation is fully reversible. The results establish macromolecular supramolecular mechanophores as a general strategy for imparting reversible mechanochromism to hydrogels.

## Graphical abstract

A supramolecular loop‐forming mechanophore featuring a quencher and an emitter is incorporated as a water‐soluble macromolecular additive into hydrogels. In the stress‐free state, intramolecular charge‐transfer interactions fold the loop and quench fluorescence. Mechanical force unfolds the loop, disrupting the quenching process and activating fluorescence. Upon release of the force, the loop refolds, and the quenched state is restored, enabling fully reversible, non‐covalent mechanochromism in water‐rich hydrogel matrices.

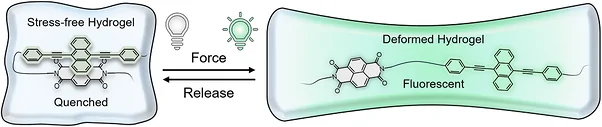

## 1 Introduction

The covalent incorporation of mechanochromic mechanophores into polymers has enabled the development of materials that visualize mechanical stress and deformation in real time [1]. In these molecular motifs, force‐induced chemical reactions [2], conformational changes [3], or the spatial separation of electronically interacting motifs [4, 5] are harnessed to generate optical signals, most commonly changes in absorption or fluorescence. Different mechanophores have been covalently integrated into a wide range of polymer architectures, including elastomers, thermoplastics, fibers, and composites, enabling the visualization of stress distributions, damage accumulation, and failure processes in otherwise optically silent materials [6]. The adaptation of the mechanophore concept to hydrogels is of considerable interest because these hydrated materials are widely used as soft functional materials [7] in areas ranging from tissue engineering [8] and soft robotics [9] to electronic and wearable devices [10] and stimuli‐responsive systems [11]. However, compared to the extensive body of work on bulk polymers [12], mechanophore‐containing hydrogels remain much less explored. A recent review by Soares and Besenius provides an excellent summary of the field [13]. Most of the mechano‐responsive hydrogels reported to date rely on covalently integrated mechanophores that undergo reversible isomerization reactions and change their absorptions and/or fluorescence characteristics, notably spiropyrans [14, 15], or xanthenes [16]. Several researchers have also used mechanophores that undergo irreversible covalent bond scission, such as the chemiluminescent bis(adamantyl)‐1,2‐dioxetane motif [17], and Diels–Alder adducts, whose activation yields photoluminescent products [18].

To overcome limitations associated with the relatively high activation forces of covalent mechanophores and their limited or slow reversibility, increasing attention has recently been directed to supramolecular mechanophores [19]. These molecules rely on the mechanically induced dissociation of optically active motifs that display weak noncovalent interactions, such as quencher‐emitter pairs [20], excimer‐forming emitters [21, 22], energy‐transfer pairs [23], and charge‐transfer complexes [24]. The weak interactions underlying these motifs [25] typically yield low activation forces and rapid, reversible responses. For instance, single‐molecule force spectroscopy studies afforded dissociation forces on the order of 100 pN for a loop‐forming mechanophore based on two perylene diimide dyes [25]. Several mechanochromic hydrogels bearing covalently attached chromophoric side chains have been reported, but examples incorporating well‐defined supramolecular mechanophores with ground‐state interactions remain limited. For instance, Rasch and Göstl used pyrene‐based macrocross‐linkers whose pendant pyrene units form deformation‐dependent excimers, enabling reversible mechanochromism in hydrogel networks [26]. Walther and coworkers used a FRET process to create tunable DNA tension probes [27], while Sagara and coworkers incorporated a rotaxane‐based supramolecular mechanophore displaying a reversible fluorescence turn‐on into a polyurethane‐urea matrix to form a mechanochromic hydrogel [23]. Both systems exhibit pronounced mechanochromic effects, but the synthesis of the mechanophores is complex, and these motifs must be covalently incorporated into the hydrogel network, complicating material preparation and limiting modularity.

To address these challenges, we here report a strategy in which a supramolecular mechanophore is transformed into a water‐soluble macromolecular additive that can be incorporated into hydrogels without covalent integration. The concept is demonstrated using a loop‐forming mechanophore (**Loop**) comprising a naphthalene diimide (NDI) quencher and a 9,10‐bis(phenylethynyl)anthracene (BPEA) fluorophore connected by a short spacer (Figure 1). The design is inspired by previous supramolecular mechanophores, notably loop‐forming motifs [28, 29], rotaxanes, and cyclophanes based on quencher‐emitter pairs [24, 30]. In the stress‐free state, intramolecular charge‐transfer (CT) interactions promote loop folding and quenching of the BPEA emission. Mechanical deformation unfolds the motif and restores BPEA emission. The functionalization of **Loop** with poly(oligo(ethylene glycol) methyl ether methacrylate) (pOEGMA) yields the water‐soluble additive **pOEGMA‐Loop**, which can be physically incorporated into various hydrogel matrices.

**FIGURE 1:**
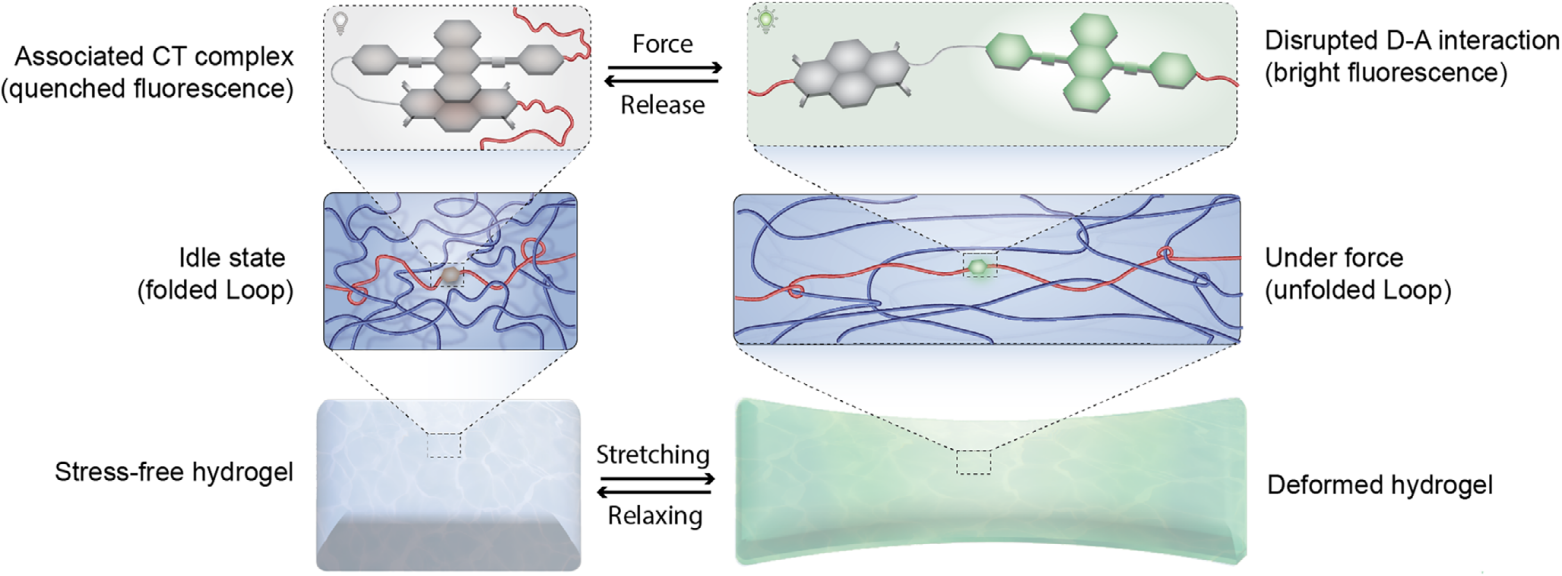
Schematic representation of the operating principle of the macromolecular mechanochromic additive. The supramolecular loop‐forming mechanophore is incorporated near the center of a water‐soluble macromolecule. In the stress‐free state, intramolecular charge‐transfer (CT) interactions between quencher and emitter moieties promote a folded loop conformation, and the emission is quenched. The application of mechanical force unfolds the Loop, and the emission is restored. The additive can be blended into a hydrogel of choice, and the mechanophores activate upon deformation.

## 2 Results and Discussion

Based on the well‐established charge‐transfer interactions of NDI‐containing donor‐acceptor systems [31, 32], we speculated that electron‐poor NDI (acceptor, A) and highly fluorescent BPEA [33] (donor, D) could serve as a weakly interacting quencher‐emitter pair for loop‐forming supramolecular mechanophores. Indeed, the NDI–BPEA pair was recently exploited in a catenane mechanophore, in which two NDI residues were combined to quench the fluorescence of a BPEA residue [34]. Our synthesis of a loop‐based supramolecular mechanophore based on this pair, shown in Scheme 1, followed a convergent approach. Asymmetric NDI acceptor (**A2**) and BPEA donor (**D2**) building blocks were prepared separately by adapting established protocols [35, 36]. Each building block was equipped with an *α*‐bromoisobutyrate ester group that can serve as an atom transfer radical polymerization (ATRP) initiator [37]. Subsequent coupling furnished the **D‐A Loop** mechanophore. Since this motif features initiators at both termini, ATRP can be used to incorporate it near the center of macromolecules, whose structures can be widely varied via monomer selection. Intermediates **A1** and **D1** were used as low‐molecular‐weight model compounds and were also further functionalized with a second *α*‐bromoisobutyrate ester to prepare NDI‐only and BPEA‐only reference additives (Schemes S4 and S5).

**SCHEME 1:**
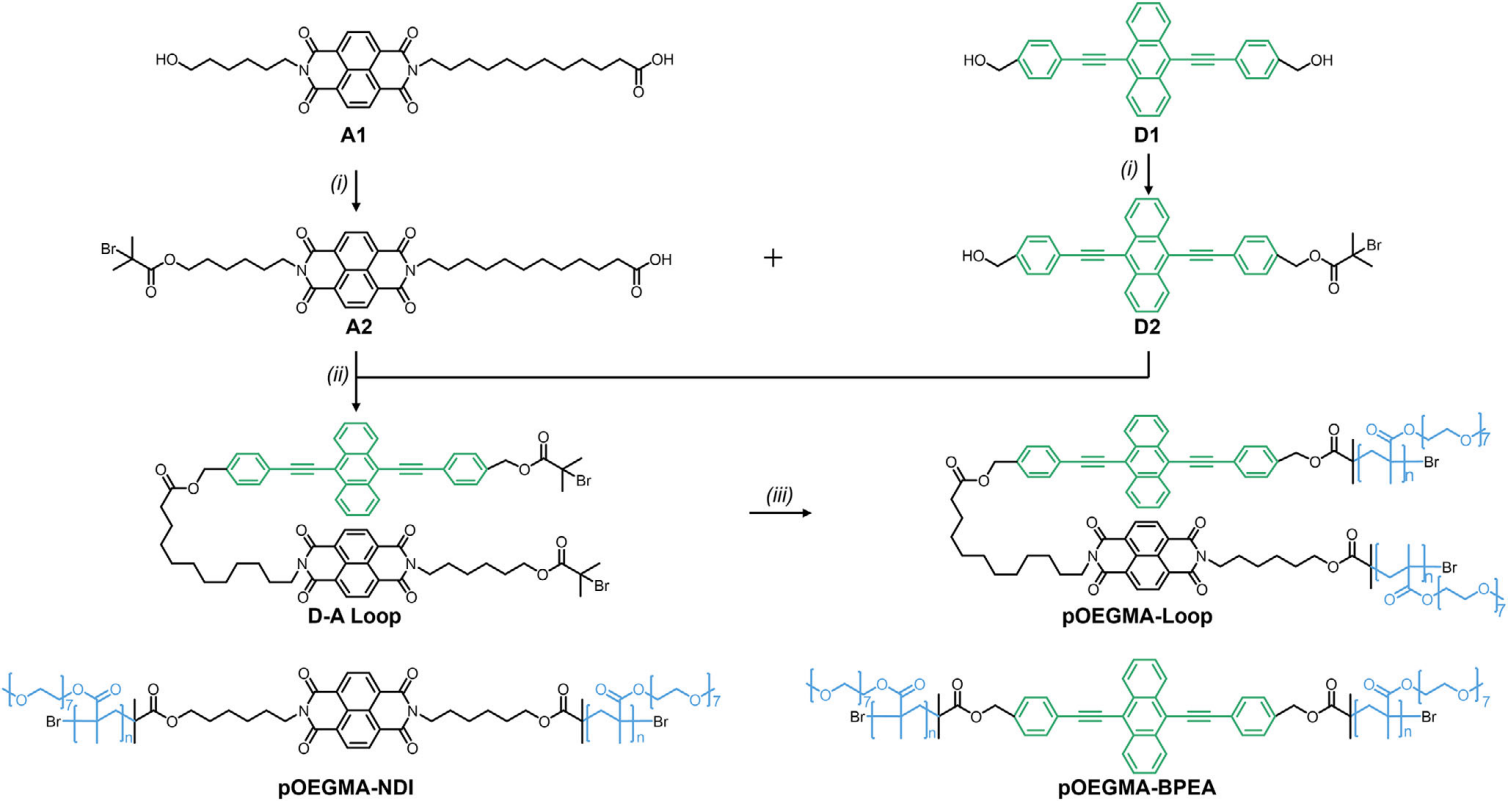
Convergent synthesis of the loop‐containing water‐soluble macromolecular mechanochromic additive **pOEGMA‐Loop**. Reagents and conditions: (i) *α*‐Bromoisobutyryl bromide, triethylamine, THF, 0°C‐—r.t, 18 h; (ii) EDC·Cl, DMAP, THF, r.t., 18 h; (iii) OEGMA_500_, CuBr_2_, PMDETA, DMSO, r.t., 2 h; (iii) OEGMA_500_, CuBr_2_, PMDETA, DMSO. Also shown are the structures of the reference additives **pOEGMA‐NDI** and **pOEGMA‐BPEA**; their syntheses are shown in Scheme S1.

Water‐soluble pOEGMA was grown from the hydrophobic initiators via ATRP of OEGMA_500_ with an average of 7 ethylene glycol units. pOEGMA was selected because its hydrophilic comb‐like architecture affords excellent water solubility, minimizes aggregation of the hydrophobic mechanophore core, and was expected to permit the mechanical coupling of the additive to the surrounding hydrogel matrix [38, 39]. While pOEGMAs exhibit lower critical solution temperature (LCST) behavior, monomers with 7–9 ethylene glycol units afford materials that display an LCST of ca. 90 °C and are water‐soluble below this temperature [40]. ATRP with **D‐A Loop** as initiator afforded **pOEGMA‐Loop** with a number‐average molecular weight (*M*_n_) of 35 kg mol^−1^ and a dispersity *(Đ)* of 2.3. We also prepared reference polymers containing only the NDI quencher (**pOEGMA‐NDI**, *M*_n_ = 32 kg mol^−1^, *Đ* = 2.3) and only the BPEA emitter (**pOEGMA‐BPEA**, *M*_n_ = 43 kg mol^−1^, *Đ* = 2.5) in a similar manner. The **pOEGMA** additives exhibit higher dispersities than the neat **pOEGMA** polymerized via ATRP under optimized conditions [41]. This reduced control is likely related to the relatively low monomer‐to‐initiator ratios, the deliberate use of mild polymerization conditions to protect the **D‐A Loop** initiator, and the limited solubility of the latter under the chosen conditions. All polymers were characterized by ^1^H NMR spectroscopy (Figures S12 and S22) and size‐exclusion chromatography (SEC) (Figure S1).

The optical properties of solutions of the low‐molecular‐weight Loop initiator **D‐A Loop** and the NDI (**A1**) and BPEA (**D1**) model compounds (*c*  =  10 µmol L^−1^) were characterized by UV‐vis absorption and photoluminescence (PL) spectroscopy in THF. The absorption spectra show bands below ca. 400 nm (**A1**) and between ca. 350–490 nm (**D1**), which display the characteristic vibronic fine structures that are diagnostic of the respective chromophores (Figure 2a). The absorption spectrum of **D‐A Loop** is largely a superposition of these spectra; however, in contrast to a mixture of the model compounds, the relative intensities of the vibronic bands associated with BPEA at 440 and 465 nm change, and the spectrum shows a long tail at the low‐energy end. These features are characteristic of CT formation [31, 34], and evidence that connecting the NDI and BPEA motifs into the **D‐A Loop** structure increases the driving force for intramolecular interactions.

**FIGURE 2:**
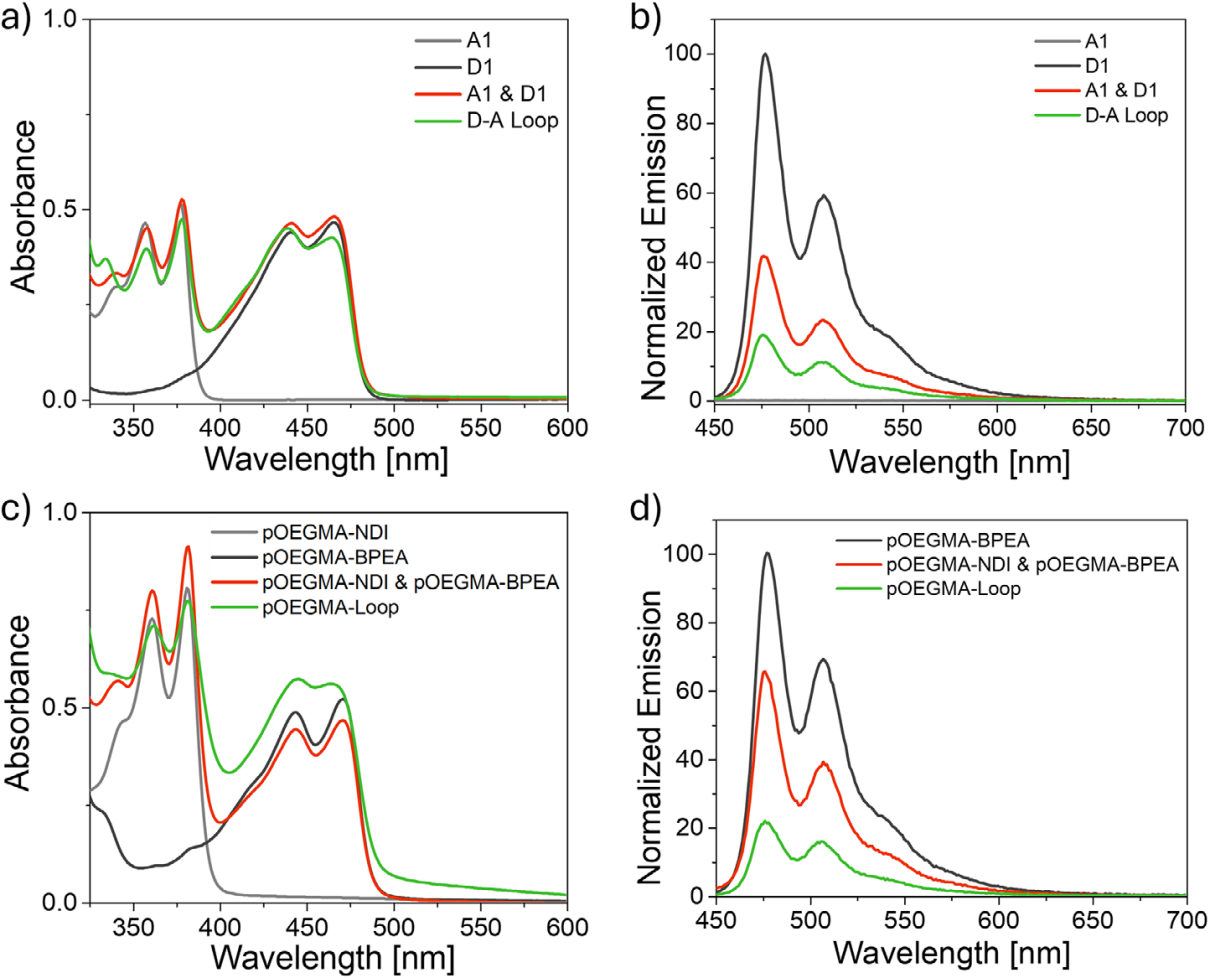
(a) Absorption and (b) PL spectra of the loop‐forming initiator **D‐A Loop**, NDI (**A1**) and BPEA (**D1**) model compounds, and an equimolar mixture of **A1** and **D1** (*c*  =  10 µmol L^−1^) in THF. (c) Absorption and (d) PL spectra of the mechanochromic additives **pOEGMA‐BPEA**, **pOEGMA‐Loop**, and an equimolar mixture of **pOEGMA‐NDI** and **pOEGMA‐BPEA** in water. The additive concentrations correspond to dye concentrations of *c*  =  10 µmol L^−1^. All PL spectra were recorded with an excitation wavelength of *λ*_ex_ = 410 nm and are normalized relative to the intensity of **D1** at 478 nm, which was set to 100%; non‐normalized spectra are shown in Figure S4.

The PL spectrum of the BPEA model compound (**D1**) in THF shows green emission and a vibronic structure with peaks at 478, 508, and 544 nm, which are characteristic of BPEA fluorescence (Figure 2b) [34]. Upon addition of an equimolar amount of the NDI‐model compound **A1**, the emission intensity drops to ca. 42% due to intermolecular quenching. The quenching is more pronounced for **D‐A Loop**, where the BPEA emission is reduced to ca. 20%.

The PL measurements of the emitter **D1**, equimolar mixtures of quencher **A1** & emitter **D1**, and the **D‐A Loop** were repeated for a range of concentrations (0.625–10 µM) to confirm that in the **D‐A Loop** intramolecular quenching occurs (Figures S5 and S6). For the **D1/A1** mixture, the BPEA fluorescence intensity increases from approximately 42% to 86% of that measured for **D1** alone at the corresponding concentration as the concentration decreases from 10 to 0.6 µM, consistent with reduced intermolecular quenching upon dilution. In contrast, the relative emission intensity of **D‐A Loop** is concentration‐independent, at ca. 20% relative to **D1**, consistent with a quenching process dominated by intramolecular emitter‐quencher interactions (Figures S5 and S6).

The photophysical characterization of the macromolecular additives was performed in water. The additive content (0.3–0.4 mg mL^−1^) was chosen so that the NDI and BPEA residue concentration matches that of the model studies (*c*  =  9–10 µmol L^−1^). Figure 2c,d shows that the optical properties of the additives in water are virtually identical to those of the model compounds in THF. Most importantly, the emission intensity of **pOEGMA‐Loop** is reduced to ca. 21% of that of **pOEGMA‐BPEA**, that is, intramolecular quenching arising from loop folding is also effective in aqueous solutions of the additive. On the other hand, a mixture of **pOEGMA‐BPEA** and **pOEGMA‐NDI** retains 65% of the emission intensity of the **pOEGMA‐BPEA** solution, slightly higher than the model compound mixture (42%), suggesting that the pOEGMA backbone reduces intermolecular interactions as intended.

We next incorporated **pOEGMA‐Loop**, **pOEGMA‐BPEA**, and **pOEGMA‐NDI** separately, and **pOEGMA‐BPEA** and **pOEGMA‐NDI** also in combination, into cross‐linked polyacrylamide (pAAm)‐based hydrogels. This hydrogel platform was selected as a first testbed due to its widespread use in biomedical and materials applications [42, 43]. Thus, acrylamide (AAm, 20 wt/v% relative to the precursor solution), N,N'‐methylenebisacrylamide (MBA, 0.05 wt/v%), water, and the respective additive or additive mixture (0.03 wt/v%) were combined to form homogeneous solutions, before the redox initiator pair ammonium persulfate (APS) and tetramethyl ethylenediamine (TEMED) was added, and the mixtures were cast into molds. In all cases, self‐supporting, elastic hydrogels formed within approximately 10 min (Figure 3a). We refer to these hydrogels as **pAAm‐Loop**, **pAAm‐NDI**, **pAAm‐BPEA**, and **pAAm‐NDI‐BPEA**. To assess whether chain‐transfer reactions to the additive might alter its structure or lead to covalent incorporation into the hydrogel, a control experiment was performed in which acrylamide was polymerized in the presence of **pOEGMA‐Loop**, without the MBA cross‐linker (Page S10). The linear **pAAm** and the additive were subsequently separated by selective precipitation (Figure S3). The SEC traces of the recovered **pOEGMA‐Loop** (Figure S2) are comparable to those of the pristine additive (Figure S1) and provide no indication of significant chain‐transfer.

**FIGURE 3:**
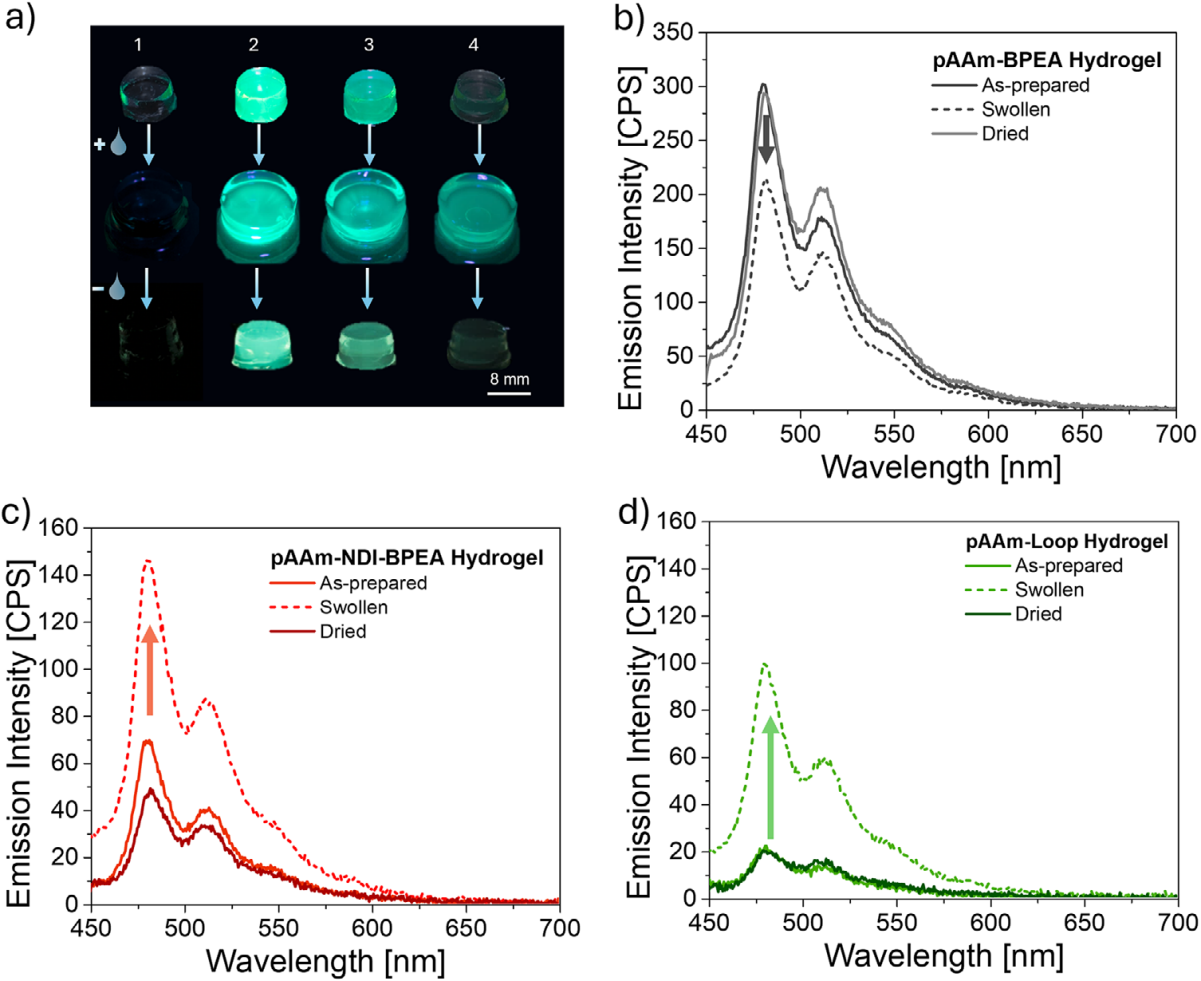
a) Photographs of **pAAm‐NDI** (1), **pAAm‐BPEA** (2), **pAAm‐NDI‐BPEA** (3), and **pAAm‐Loop** (4) hydrogels. Shown are as‐prepared gels (top), gels swelled in water to 700% of their original weight (middle), and gels that were dried after swelling to their initial weight (bottom). All samples were irradiated with UV light (*λ*_ex_ = 395 nm) at the same intensity, but the brightness of the three rows was adjusted to match perception. b,c,d) PL spectra of the as‐prepared and water‐swollen (700%) and redried **pAAm‐BPEA** (b), **pAAm‐NDI‐BPEA** (c), and **pAAm‐Loop** (d) gels. The spectra were collected at *λ*_ex_ = 405 nm.

The materials produced are structurally analogous to semi‐interpenetrating polymer networks (semi‐IPNs), a well‐established class of materials that combine a cross‐linked host network with physically entrapped linear guest polymers [44]. The concentration of **pOEGMA‐Loop** (0.03 wt/v%) is well below the overlap concentration expected for flexible polymers of comparable molar mass, rendering entanglements between **pOEGMA‐Loop** chains highly unlikely. Consequently, stress transfer must arise from physical confinement and topological trapping of **pOEGMA‐Loop** chains within the heterogeneous pAAm network [45]. This interpretation is consistent with the molecular weight of the **pOEGMA‐Loop** (*M*_n_ = 35 kg mol^−1^) and the nominal molecular weight between cross‐links of the pAAM network (*M*_c_ = approximately 31 kg mol^−1^, estimated from the AAm/MBA feed composition, Page S20). While these values place the **pOEGMA‐Loop** additive and network strands on comparable macromolecular length scales and are therefore consistent with topological trapping, they cannot be directly compared to quantitatively assess entanglement density or the degree of confinement.

The pictures shown in Figure 3a and the corresponding emission spectra (Figure 3b,c,d) demonstrate that the four hydrogels exhibit PL characteristics consistent with those observed in solution. The **pAAm‐NDI** gel does not show any emission (Figure S7), while the quencher‐free **pAAm‐BPEA** gel exhibits characteristic BPEA emission and features the highest emission intensity among all as‐prepared gels (Figure 3a,b). The **pAAm‐NDI‐BPEA** and **pAAm‐Loop** gels show slightly and strongly quenched emission, respectively (Figure 3a,c,d). The gels were then swelled to equilibrium in water, which led to a relative mass increase of S = (*m*_eq_‐*m*_0_)/*m*_0_ = 700%, where *m*_0_ and *m*_eq_ are the masses of the freshly prepared and the fully swollen gels, respectively. As a result, the emission intensity of the **pAAm‐BPEA** hydrogel decreased by ca. 30% due to dilution of the fluorophore in the swollen network (Figure 3b). In contrast, the emission intensities of the **pAAm–NDI–BPEA** and **pAAm–Loop** gels increase 2.1‐fold and 4.8‐fold, respectively, indicating that the reduction in quenching upon swelling outweighs the decrease in emitter concentration. In the case of the **pAAm–NDI–BPEA** gel, this effect must be related to an increase in the average intermolecular distance between the **pOEGMA‐NDI** and **pOEGMA‐BPEA** additives [46], whereas in the **pAAm–Loop** gel, the Loop motif unfolds. The Loop design yields a substantially higher emission‐intensity ratio between the stretched and unstretched states. The swelling‐related optical changes are reversible, that is, the original emission intensity is restored upon drying to the as‐prepared water content (Figure 3a).

The absorption spectra (Figure S8) and the visual appearance of the gels (Figure S9) further support the presence of donor–acceptor interactions. While the characteristic BPEA vibronic bands are observed in all BPEA‐containing gels, the as‐prepared **pAAm–Loop** gel exhibits less resolved vibronic structure and a more pronounced long‐wavelength absorption than the **pAAm–NDI–BPEA** control, consistent with stronger intramolecular charge‐transfer interactions. Swelling sharpens the vibronic structure and reduces the long‐wavelength absorption, suggesting that expansion of the hydrogel network weakens the donor–acceptor interactions.

The response of the various as‐prepared hydrogels to mechanical deformation was explored by uniaxial deformation of all BPEA‐containing gels to a strain (ε) of 430% and recording the emission intensity in situ (Figure 4). All gels exhibit nearly identical stress–strain behavior compared to the neat **pAAm** hydrogel reference (Figure S10), confirming that incorporating the different additives does not affect the mechanical properties of the hydrogel networks (Figure 4b,c). The stress‐strain curves are largely linear, with slight strain hardening at strains above ca. 350%. Upon deformation, the fluorescence intensity of the **pAAm–BPEA** control, measured at 478 nm, displays a gradual decrease from ca. 300 counts per second (CPS) in the undeformed state to ca. 120 CPS at *ε* = 430% (Figure 4c), which is primarily due to sample thinning upon elongation. The **pAAm–NDI–BPEA** gel exhibits a substantially lower initial fluorescence intensity (ca. 60 CPS), which increases approximately twofold to ca. 120 CPS upon stretching, indicating progressive turn‐off of the NDI‐BPEA interactions. The **pAAm–Loop** gel exhibits the lowest fluorescence intensity in the stress‐free state (ca. 20 CPS), reflecting efficient intramolecular quenching within the loop‐forming motif. In this case, the fluorescence intensity increases prominently with strain, reaching ca. 120 CPS at *ε* = 430% (Figure 4c). Note that at *ε* = 430%, the three compositions show comparable emission intensities, which reflects that in the **pAAm–NDI–BPEA** and **pAAm–Loop** gels, the quencher‐emitter interactions are completely switched off (Figure 4b,c). The close correspondence between the stress–strain and emission intensity–strain traces indicates that deformation of the poly(acrylamide) network is coupled to separation of the donor and acceptor units in the embedded additives. Importantly, the ratio of the emission intensities in the idle and stretched (*ε* = 430%) state is substantially higher for the **pAAm–Loop** (sixfold) than for the **pAAm–NDI–BPEA** gel (twofold). This larger contrast arises from the strong intramolecular quenching provided by the folded Loop in the unstretched state, together with its force‐induced unfolding upon deformation. A macroscopic visualization of this fluorescence turn‐on of a **pAAm‐Loop** gel under uniaxial deformation is provided in Movie S1.

**FIGURE 4:**
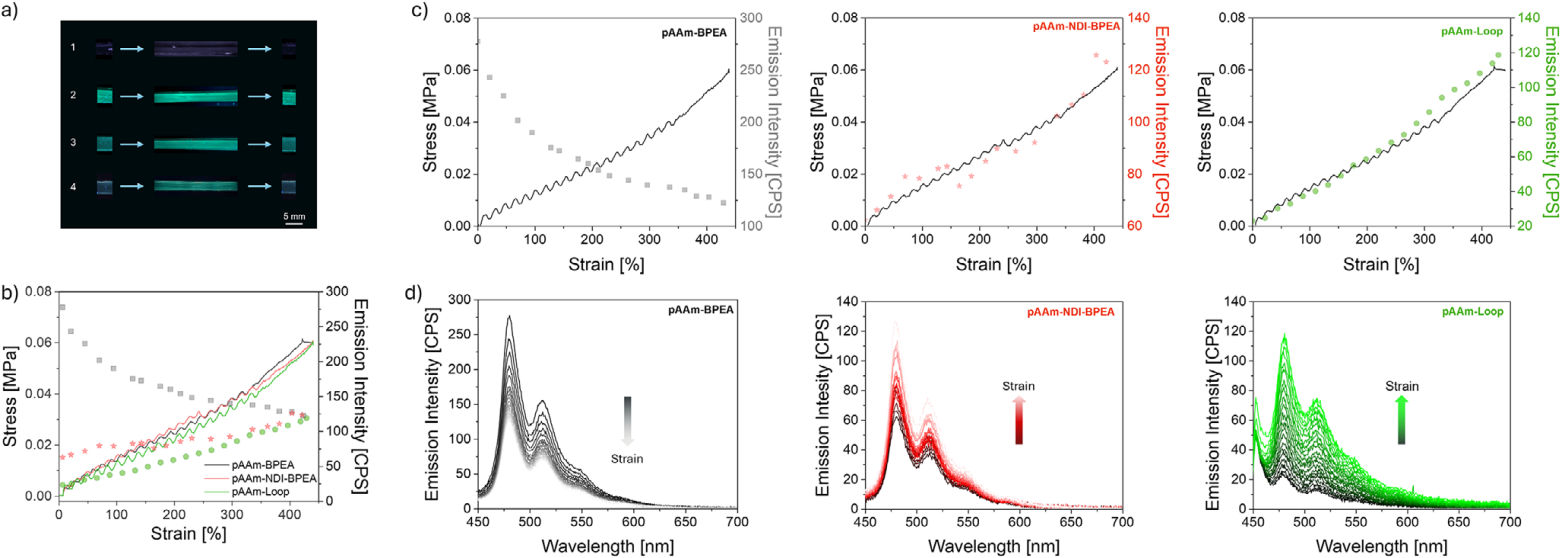
(a) Photographs (portions) of **pAAm‐NDI** (1), **pAAm‐BPEA** (2), **pAAm‐NDI‐BPEA** (3), and **pAAm‐Loop** (4) gels in the stress‐free state (left), stretched to 430% (middle), and relaxed after stretching (right). All samples were irradiated with UV light (*λ*_ex_ = 395 nm) of the same intensity. (b) Overlay of the stress–strain curves (solid lines) and the emission (*λ*_ex_ = 405 nm) intensities at 478 nm (symbols) recorded during stretching of the **pAAm‐BPEA, pAAm‐NDI‐BPEA**, and **pAAm‐Loop**) hydrogels. (c) Overlay of the normalized stress–strain curves (solid lines) and the emission intensity at 478 nm (symbols). d) PL spectra recorded upon stretching of the **pAAm‐BPEA**, **pAAm‐NDI‐BPEA,** and **pAAm‐Loop** hydrogels to a strain of *ε*  = 430%.

Figures 5a and S11a show that the activation of **D‐A Loop** in the **pAAm‐Loop** gel is fully reversible, that is, the intensity increases and decreases over 12 stretch‐and‐release cycles (*ε*  = 0–430%) without any appreciable hysteresis. A similar behavior is observed under cyclic compression (Figure S11b,c), confirming that the force transfer to the additive is effective under both tensile and compressive deformation. The compressive strain is limited to ca. 68%, and consequently, the attainable intensity change (ca. 20 to 40 CPS) is smaller than that reached upon stretching.

**FIGURE 5:**
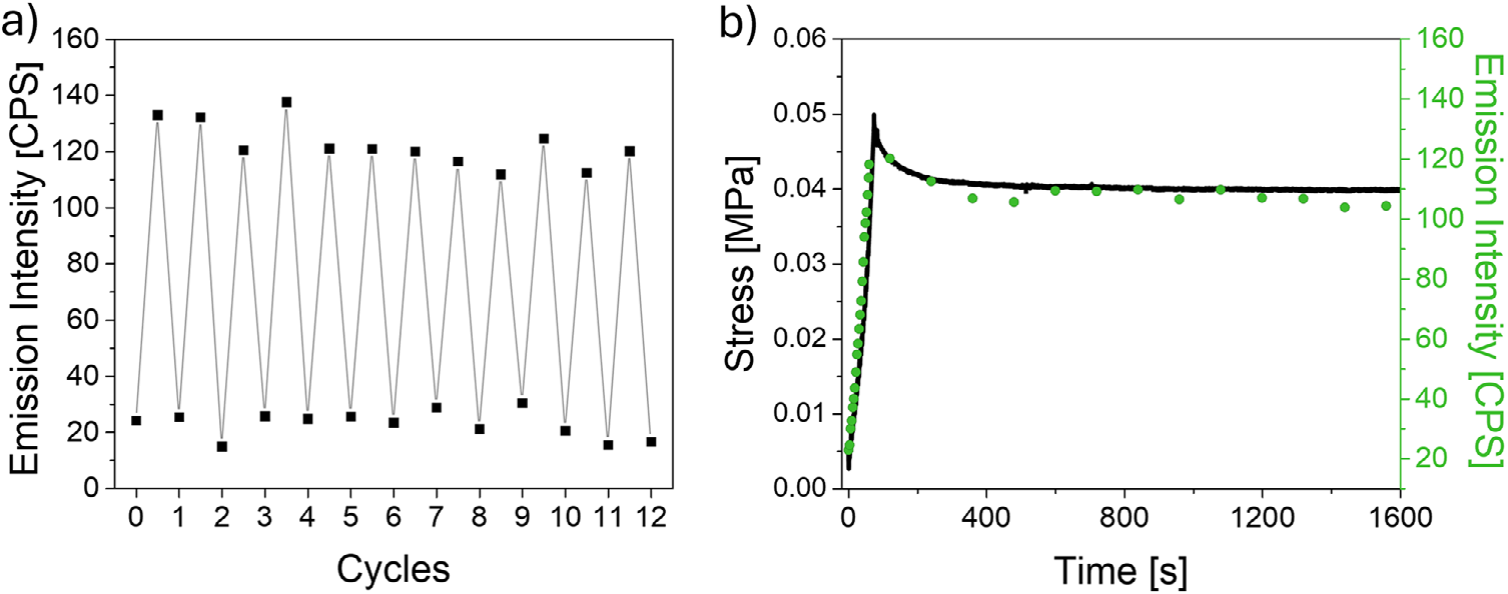
a) Mechanoresponse of a **pAAm‐Loop** gel upon cyclic stretching. The plot shows the emission intensity of the gel in force‐free and uniaxially stretched (*ε* = 430%) states, recorded over 12 stretching‐relaxation cycles. (b) Stress relaxation experiment. The plot shows an overlay of the stress–strain and emission intensity traces recorded upon stretching a **pAAm‐Loop** gel to a strain of *ε* = 380%, fixing the strain, and recording the stress and emission intensity over 1600 sec. In all experiments, the emission intensity was recorded at 478 nm with *λ*_ex_ = 405 nm.

We also carried out a stress‐relaxation experiment in which the **pAAm‐Loop** gel was uniaxially deformed to a strain of 380% and then kept under strain, with stress and emission intensity (Figures 5b and S12) monitored over time. The applied stress shows only a small initial drop in stress before reaching a stable plateau. Gratifyingly, the emission intensity evolves in parallel with the stress throughout the experiment, indicating that the mechano‐response is coupled to the time‐dependent relaxation of the hydrogel network.

To quantify the extent of mechanophore activation, the switched‐on fraction (*f*_ON_) was estimated from the strain‐induced fluorescence of the **pAAm–Loop** gel relative to the **pAAm–BPEA** control (Page S19). The activated fraction increases continuously with strain, reaching 0.46 at approximately 286% strain and 0.97 at 430% strain (Table S3). To demonstrate the generality of the approach, the **pOEGMA–Loop** additive was also incorporated into pOEGMA‐based hydrogels by free‐radical polymerization of OEGMA_500_ using poly(ethylene glycol) dimethacrylate (*M*_n_ = 750 g mol^−1^) as cross‐linker under conditions analogous to those employed for the pAAm hydrogels (see Supporting Information for experimental details). Gratifyingly, the mechanochromic responses of the pOEGMA hydrogels are very similar to those of the **pAAm‐Loop** gel (Figure S13). Swelling to equilibrium induces a pronounced fluorescence turn‐on, while tensile deformation results in efficient fluorescence activation through force‐induced disruption of the BPEA–NDI charge‐transfer interaction. Cyclic compression likewise produces a reversible increase in fluorescence. These results indicate that the additive approach reported here is generalizable and should be applicable to other hydrogels and to alternative (supramolecular) mechanophores.

## 3 Conclusion

In summary, we have developed a supramolecular loop mechanophore based on reversible charge‐transfer interactions between BPEA and NDI. The loop architecture promotes efficient quenching of BPEA fluorescence in the force‐free folded state, whereas mechanical force separates the BPEA and NDI moieties, restoring emission. The functionalization with pOEGMA affords a water‐soluble mechanochromic additive that can be readily blended into different hydrogels, enabling visualization of swelling‐ and force‐induced activation through a pronounced fluorescence turn‐on response. Comparison with intermolecular and fluorophore‐only control systems confirms that the observed emission changes predominantly originate from the disruption of intramolecular BPEA–NDI charge‐transfer interactions. Quantitative analysis reveals nearly complete mechanophore activation at high strain, while cyclic deformation experiments show reversible activation with little change in the optical response over repeated cycles. The observation of similar behavior in both pAAm‐ and pOEGMA‐based hydrogels highlights the generality of the approach and suggests broad applicability across soft polymeric materials. These findings highlight the suitability of loop‐forming donor–acceptor motifs as supramolecular mechanophores for monitoring deformation in soft, water‐rich materials. Importantly, efficient force transduction does not require covalent attachment of the mechanophore to the network, provided that the motif‐bearing polymer is physically entrapped within the heterogeneous hydrogel network, allowing macroscopic deformation to be transmitted to the supramolecular loop. The high apparent switched‐on fraction of pOEGMA‐Loop at high strain indicates that force transfer is not restricted to only the highest‐molar‐mass fraction of the **pOEGMA‐Loop** population and reflects that a substantial fraction of the loop chains is physically constrained within the heterogeneous pAAm network.

## Author Contributions

**Iulia Scarlat**: investigation, writing – original draft, methodology, validation, visualization, formal analysis, data curation. **Jess M. Clough**: conceptualization, writing – review and editing, formal analysis, supervision, methodology, validation. **Christoph Weder**: conceptualization, funding acquisition, writing – original draft, validation, methodology, visualization, writing – review and editing, formal analysis, project administration, supervision, resources.

## Conflicts of Interest

The authors declare no conflicts of interest.

## Supporting information




**Supporting File 1**: anie74622‐sup‐0001‐SupMat.docx.

**Supporting File 2**: anie74622‐sup‐0002‐VideoS1.mp4.

## Data Availability

The data that support the findings of this study are available for download at the Zenodo repository at https://doi.org/10.5281/zenodo.20446443.
