# Supplementary material for: Supramolecular Mechanophore Additives Enable Mechanochromic Hydrogels
Source: Angew Chem Int Ed Engl. 2026 Sep 7;65(44):e7699472. doi: 10.1002/anie.7699472 (PMC13703912; doi:10.1002/anie.7699472)
Supplement: Supplementary file 1 [file ANIE-65-e7699472-s002.docx]

**Supporting Information.**

**Supramolecular Mechanophore Additives Enable Mechanochromic Hydrogels**

Iulia Scarlat^[a,b],^ Jess M. Clough*^[a,b],^ and Christoph Weder*^[a,b]^

[a] Adolphe Merkle Institute (AMI), University of Fribourg, Chemin des Verdiers 4, 1700 Fribourg, Switzerland

[b] NCCR Bio-inspired Materials. University of Fribourg, Chemin des Verdiers 4, 1700 Fribourg, Switzerland

*To whom correspondence should be addressed: jessica.clough@unifr.ch, christoph.weder@unifr.ch

| **Table of contents** |  |
| --- | --- |
| **General Methods** | **S2-S3** |
| **Synthesis of A1 and A2** | **S4** |
| **Synthesis of D1 and D2** | **S4-S5** |
| **Synthesis of D-A Loop and pOEGMA-Loop** | **S5-S6** |
| **Synthesis of pOEGMA-NDI** | **S6-S7** |
| **Synthesis of pOEGMA-BPEA** | **S7-S8** |
| **Preparation of pAAm Hydrogels** | **S8-S9** |
| **Preparation of pOEGMA Hydrogels** | **S9** |
| **Probing the Inertness of pOEGMA-Loop during the Polymerization of pAAm** | **S10** |
| **Swelling Experiments** | **S10** |
| **Tensile Deformation Experiments** | **S11** |
| **Compression Experiments** | **S12** |
| **Supplementary Figures S1-S13** | **S13-S18** |
| **Calculations and Tables S2-S3** | **S19-S20** |
| **NMR Data** | **S21-26** |
| **Supporting Reference** | **S27** |

**General Methods**

All reagents and solvents were purchased from Merck, Sigma-Aldrich, Fisher Scientific, TCI, BDL Pharma, or Flurochem and were used without further purification unless otherwise stated. Residual inhibitors in OEGMA were removed by passage through basic alumina prior to polymerization. Flash silica gel column chromatography was carried out using a Biotage® Selekt system, with dry loadings onto Biotage® Sfär Silicax. Analytes were monitored by UV detection at their respective absorbance wavelengths. Microwave-assisted reactions were performed using a Biotage® Initiator 8 EXP microwave system equipped with an Initiator Robot 8, with temperature and pressure control. Reactions were carried out in sealed microwave vials under magnetic stirring. The reaction mixtures were heated to the desired temperature and maintained at this temperature for the specified time under automatic power adjustment. After completion, the reaction vials were removed and cooled to room temperature in an ice bath.

**Nuclear Magnetic Resonance (NMR) Spectroscopy**. ^1^H NMR spectra were recorded on a Bruker Avance DPX 400 spectrometer at 400 MHz. All chemical shifts (δ) of ^1^H NMR spectra are reported on the *δ*-scale in ppm relative to the signal of tetramethylsilane (TMS, at 0.00 ppm) or residual solvent protons as an internal standard. Data analysis was conducted using MestReNova (v 11.0). Multiplicities are reported as follows: s (singlet), d (doublet), dd (doublet of doublets), t (triplet), q (quintet), m (multiplet), and br (broad).

**Electrospray ionization mass spectrometry (ESI–MS).** ESI-MS measurements were performed on a Bruker Esquire HCT instrument. Spectra were recorded in positive ionization mode over a mass range of m/z 400–2000. Samples were prepared in appropriate solvents (acetonitrile or THF) concentration of ca. 50 µg mL^-1^ and analyzed via continuous flow injection (2 µL min^-1^).

**Size Exclusion Chromatography (SEC).** SEC was performed on an Agilent 1260 Infinity II HPLC system equipped with one Agilent PolarGel M guard column (8 μm) and two Agilent PolarGel M columns (7.5 mm × 300 mm, 8 μm). Samples (1.3 mg mL^-1^) were eluted at 50 °C in DMF with 0.05 M LiBr at 1.0 mL min^-1^. Detection was achieved via refractive index detector (G7162A RID) and variable wavelength detector (G7114A VWD). Molecular weights were determined using poly(ethylene oxide) calibration standards.

**Ultraviolet Visible (UV-Vis) Absorption Spectroscopy.** Electronic absorption spectra were acquired at ambient temperature using a Shimadzu UV-2401 PC spectrophotometer. Liquid samples were measured in quartz cuvettes with a 0.5 cm optical path length, fast scan speed, and a 5 nm slit width. The corresponding pure solvent was used as a reference. Hydrogel samples were measured in single-use PMMA cuvettes with a 1 cm optical path length. A hydrogel without any additive was used as a reference.

**Photoluminescence (PL) Spectroscopy**. PL spectroscopy in solution was carried out with a Horiba Fluorolog 3 spectrometer with right-angle illumination equipped with a 450 W Xenon light source for excitation and an FL-1030-UP photomultiplier as detector. Spectra were recorded with an excitation wavelength (λ_ex_) of 410 nm and an emission range of 450-700 nm with a 1 nm entrance and exit slit width. Samples were measured in quartz cuvettes with a 0.5 cm optical path length. All spectra were corrected for the detector nonlinearity.

***In* *situ* PL Spectroscopy**. Steady-state photoluminescence (PL) spectra of hydrogels were monitored with an Ocean Optics LS-405A LED light source with λ_ex =_ 405 nm, using an optical fiber (Ocean Optics QR230-7-XRS) connected to an Edmund Optics filter holder containing a 420 nm long-pass Edmund Optics optical filter. A second optical fiber (QP230-1-XRS) connected the sample holder and the Ocean Optics QE Pro Scientific grade Spectrometer. The spectra were acquired by placing the optical fiber at a 45°C angle, ca. 2 mm from the sample, and operating the system at 5% light intensity. The OceanView software was used to control the exposure time (100 ms) and number of scans (10), and to create scan-averaged spectra. The spectra were not corrected for the detector nonlinearity and were used without any further processing.

**Tensile Testing.** Hydrogels samples 17.2 × 5 × 2 mm (length x width × thickness) were measured at a constant strain rate of 6% s^-1^ using a Linkam TST350 microtensile stage equipped with a 20 N load cell using the LINK software. *In situ* PL spectra of hydrogel samples during tensile deformation were measured simultaneously by placing the optical fiber at an angle, at a fixed position above the hydrogel surface. The optical fiber remained stationary, measuring the central region of the sample throughout the stretching process.

**Photographs.** Photographs and videos of samples were recorded with a Sony A7 IV digital camera equipped with a Sigma 24-70mm f/2.8 DG DN II Art objective lens and a UV filter (Hoya UV-IR 82 mm) under ambient light or UV illumination (TATTU U3 UV Flashlight, 395 nm or TATTU U2 UV Flashlight 365 nm ). Camera settings: F-stop: f/9, f/18, ISO Speed: 100-800, exposure times: 1/50 sec. The pictures were adjusted in Adobe Lightroom so that the brightness shown in the pictures matches the visual perception of the researcher.

**Scheme S1**. Synthesis of **A1** and **A2**.

**Synthesis of A1 and** **A2**. A mixture of naphthalene-1,4,5,8-tetracarboxylic dianhydride (500 mg, 1.86 mmol, 1 eq.), 12-aminolauric acid (350 mg, 1.63 mmol, 0.87 eq.) and (triethyl)amine (0.25 mL, 1.79 mmol, 1.05 eq.) was dissolved in DMF (20 mL) in a 20 mL microwave vial equipped with a stir bar. The solution was pre-stirred for 1 minute and then subjected to microwave irradiation for 5 min at 140 °C. The vial was removed from the microwave reactor and placed on an ice bath 1-Amino-6-hexanol (200 mg, 1.71 mmol, 0.92 eq) was added, and the solution was again subjected to microwave irradiation for 5 min at 140 °C. The vial was removed from the microwave reactor and placed on an ice bath . The solvent was removed in vacuo, and the residue was suspended in acetone (10 mL), precipitated in 1 M aq. HCl solution (20 mL), and collected by filtration. Purification of the filtrate by flash column chromatography (silica gel, chloroform/MeOH 98:2 v/v%) yielded **A1** (580 mg; 55%) as an off-white powder.

^1^H NMR (400 MHz, DMSO-d6) δ: 11.87 (s, 1H), 8.62 (s, 4H), 4.70 (s, 2H), 3.62 (t, J = 6.2 Hz, 2H), 3.14 (t, J = 7.1 Hz, 2H), 2.21 (t, J = 7.1 Hz, 2H), 1.67–1.26 (m, 14H). Exact Mass: 564.26, found mass: 563.27 ([M-H]^-^)

Compound **A1** (350 mg, 0.60 mmol, 1 eq.) was dissolved in dry THF (50 mL), in a 100 mL flask equipped with a stir bar, and triethylamine (0.2 mL, 1.43 mmol, 2.37 eq.) was added. The solution was cooled to 0 °C on an ice bath, and $\alpha$-bromoisobutyryl bromide (150 mg, 0.65 mmol, 1.08 eq.) was added dropwise to the stirred reaction mixture over the course of 20–30 min. After the addition was complete, the reaction mixture was stirred at 0 °C for an additional 30 min, then brought to room temperature and stirred for an additional 18 h. The solvent was removed under reduced pressure and purified by flash column chromatography (silica gel, chloroform/MeOH 98:2 v/v%) to afford **A2** (240 mg, 54%) as a beige solid.

^1^H-NMR (400 MHz, CHCl_3_) δ: 8.69 (s, 4H, ArH), 4.10 (t, J = 6.0 Hz, 2H, CH_2_O), 3.69 (t, J = 6.0 Hz, 2H, CH2O), 3.07 (t, J = 6.8 Hz, 2H, CH_2_CO), 2.47 (t, J = 6.8 Hz, 2H, CH_2_Br), 1.93 (s, 6H, CH_3_), 1.65–1.26 (m, aliphatic CH_2_). Exact Mass: 712.24, found mass: 735.23 ([M+Na]^+^)

**Scheme S2**. Synthesis of **D1** and **D2**.

**Synthesis of D1 and D2.** In a glovebox, Pd(PPh₃)₂Cl₂ (200 mg, 0.28 mmol, 0.10 eq.) and CuI (30 mg, 0.16 mmol 0.05 eq.) were weighed into a 50 mL flask and dispersed in anhydrous THF (10 mL). In a separate 250 mL Schlenk flask equipped with a stir bar, 9,10-dibromoanthracene (1.00 g, 2.97 mmol, 1.00 equiv.) was dissolved in a mixture of anhydrous THF (60 mL) and dry triethylamine (15 mL), and the stirred solution was degassed with N_2_. The catalyst suspension was then added slowly to the reaction mixture under a flow of N_2_. Subsequently, a solution of 4-ethynylbenzyl alcohol (1.18 g, 9.08 mmol, 3.00 equiv.) in anhydrous THF (10 mL) was added dropwise under N_2_. Upon addition, the color of the reaction mixture changed from orange to black. The mixture was heated to 65 °C and stirred under reflux for 18 h. After cooling to room temperature, the resulting precipitate was removed by filtration. The crude product was precipitated by addition of the reaction mixture to cold hexane (500 mL), filtered off, and the solid residue thus collected was purified by flash column chromatography on silica gel (gradient to 1/1 v/v% hexanes/ethyl acetate) to afford **D1** (490 mg, 63%) as a yellow solid.

^1^H-NMR (400 MHz, CHCl_3_) δ: 8.65 (q, J = 7.9 Hz, 4H, ArH), 7.71 (d, J = 8.2, 1.5 Hz, 4H, ArH), 7.57 (q, 4H, ArH), 7.41 (m, 4H, ArH), 4.73 (s, 4H, CH_2_OH). Exact Mass: 438.16, found mass: 439.17 ([M+H]^+^)

Compound **D1** (400 mg, 0.91 mmol, 1 eq.) was dissolved in dry THF (50 mL) in a 100 mL flask equipped with a stir bar, and triethylamine (0.4 mL, 2.97 mmol, 3.15 eq.) was added. The stirred solution was cooled to 0 °C in an ice bath, and $\alpha$-bromoisobutyryl bromide (230 mg, 1 mmol, 1.1 eq.) was added dropwise over the course of 20–30 min. After the addition was complete, the reaction mixture was stirred at 0 °C for an additional 30 min, then brought to room temperature and stirred at this temperature. The progress of the reaction was monitored by TLC, and the reaction was stopped after ca. 18 h when the mono-ester **D2** was the major product. The by-product precipitate was filtered off, the solvent was removed under reduced pressure, and the solid product was purified by flash column chromatography on silica gel (gradient to 50/50 v/v% hexanes/ethyl acetate) to afford **D2** (120 mg, 23%) as a bright yellow solid.

^1^H-NMR (400 MHz, CHCl_3_) δ: 8.70 (m, J = 7.9 Hz, 4H, ArH), 7.78 (d, J = 8.2, 1.5 Hz, 4H, ArH), 7.67(q, 4H, ArH), 7.49 (m, 4H, ArH), 5.28 (s, 2H, CH_2_OH), 4.79 (s, 2H, CH_2_O), 1.99 (s, 6H, CH_3_). Exact Mass: 586.11, found mass: 587.12([M+H]^+^)

**Scheme S3.** Synthesis of **D-A Loop** and **pOEGMA-Loop.**

**Synthesis of D-A Loop and pOEGMA-Loop.** Compounds **A2** (120 mg, 0.17 mmol, 1 eq.) and **D2** (99 mg, 0.17mmol, 1 eq.), 1-ethyl-3-(3-dimethylaminopropyl)carbodiimide hydrochloride (EDCI, 75, 0.39 mmol, 2.3 eq.), and N,N′-dicyclohexylcarbodiimide (DMAP, 50 mg, 0.41 mmol, 2.4 eq.) were dissolved in a 20 mL vial equipped with a stir bar in dry THF (15 mL) under N_2_ atmosphere. The mixture was stirred at room temperature for 18 h. Afterwards, the THF was removed under reduced pressure, and the crude product was re-dissolved in DCM (20 mL). The mixture was transferred to a separatory funnel, and the organic phase was washed with saturated NaCl solution (3 x 20 mL), dried over Na_2_SO_4_, and filtered. The solvent was removed under reduced pressure, and the crude product was purified by flash column chromatography (silica gel, gradient eluent: DCM to DCM/MeOH 9:1 v/v%) to obtain **pOEGMA-Loop** (130 mg, 60%) as a red solid.

^1^H-NMR (400 MHz, CHCl_3_) δ: 8.68 (s, 2H, NDI ArH), 8.45 (s, 2H, NDI ArH), 7.71 (d, J = 7.5 Hz, 4H, ArH), 7.53 (t, J = 7.5 Hz, 4H, ArH), 7.41 (t, J = 7.5 Hz, 4H, ArH), 5.23 (s, 2H, vinyl =CH₂), 5.14 (s, 2H, vinyl =CH₂), 4.10 (m, 4H, OCH₂), 2.36 (m, 2H, CH₂), 1.93 (m, 6H, CH₂/CH₃), 1.85 (m, 4H, CH₂), 1.65 (m, 8H, CH₂). Exact Mass: 1280.34, found mass: 1281.35 ([M+H]^+^)

OEGMA was passed through a column of basic alumina. A copper wire (~10 cm) was sanded to remove the oxide surface layer, washed with acetone, and wrapped around a cylindrical stir bar. OEGMA_500_ (2.00g, 4.00 mmol, 100 eq.), **D-A Loop** (51.32 mg, 0.04 mmol, 1 eq.), Cu(II)Br_2_ (0.45 mg, 0.0025 mmol, 0.05 eq.), and DMSO (2 mL) were added to a 20 mL vial containing the stir bar wrapped with copper wire. The vial was sealed with a septum lid. To increase the solubility of **D-A Loop**, the mixture was warmed up to 35°C and sparged with N_2_ for 30 min. To start the reaction, PMDETA (0.002 mL, 0.01 mmol, 0.3 eq.) was added, and the mixture was stirred at room temperature for 2 h. The viscous mixture was diluted with acetone (5 mL) before being passed through a silica column to remove the copper complex. Excess acetone was removed under reduced pressure, and the product was purified twice by precipitation into cold diethyl ether (3 x 20 mL). The precipitate was filtered off, dried under vacuum at 60 °C to yield 0.98 g (49%) **pOEGMA-Loop** as a yellow viscous liquid.

^1^H-NMR (400 MHz, DMSO) 4.20–3.30 (m, OCH_2_CH_2_O), 3.44 (s, OCH_3_), 2.5 (m, backbone/aliphatic protons). SEC (DMF): *M*_n_ = 35 kg mol^-1^; *Đ* =2.3.

**Scheme S4.** Synthesis of **pOEGMA-NDI.**

**Synthesis of** **pOEGMA-NDI.** A mixture of naphthalene-1,4,5,8-tetracarboxylic dianhydride (400 mg, 1.49 mmol, 1 eq.), 6-amino-1-hexanol (400 mg, 3.41 mmol, 2.3 eq.) and (triethyl)amine (0.25 mL, 1.20 mmol, 1.05 eq.) was dissolved in DMF (10 mL) in a microwave vial equipped with a stir bar. The solution was subjected to microwave irradiation for 5 min at 140 °C. The solvent was removed under reduced pressure, and the residue was suspended in acetone (5 mL), precipitated in 1 M aq. HCl solution (10 mL), and filtered to afford **A3** light-pink solid (500 mg, 72%).

^1^H NMR (400 MHz, CDCl3) δ**:** 8.62 (s, 4H, ArH), 4.10 (t, J = 6.2 Hz, 2H, CH2OH), 3.62 (t, J = 6.2 Hz, 2H, CH2OH), 3.14 (t, J = 7.1 Hz, 2H, CH2N), 1.67–1.29 (m, aliphatic CH2).

Compound **A3** (170 mg, 0.36 mmol, 1 eq.) was dissolved in dry THF (20 mL) in a 50 mL flask equipped with a stir bar, and triethylamine (0.2 mL, 1.43 mmol, 2.37 eq.) was added. The solution was cooled to 0 °C in an ice bath, and $\alpha$-bromoisobutyryl bromide (200 mg, 0.87 mmol, 2.4 eq.) was added dropwise. The reaction mixture was stirred at room temperature for 18h. The solvent was removed under reduced pressure, and the product was purified by flash column chromatography (silica gel, chloroform/MeOH 98:2 v/v%) to afford **A4** (180 mg, 64%) as a beige solid.

^1^H NMR (400 MHz, CDCl3) δ: 8.62 (s, 4H, ArH), 4.13 (t, J = 6.1 Hz, 2H, OCH2), 3.14 (t, J = 7.1 Hz, 2H, CH2N), 1.67 (m, 2H, CH2), 1.60 (m, 2H, CH2), 1.43 (m, 2H, CH2), 1.29 (m, 2H, CH2), 2.02 (s, 6H, CH3).

OEGMA was passed through a column of basic alumina. A copper wire (~10 cm) was sanded to remove the oxide surface layer, washed with acetone, and wrapped around a cylindrical stir bar OEGMA_500_ (2.00g, 4.00 mmol, 100 eq.), compound **A4** (31 mg, 0.04 mmol, 1 eq.), Cu(II)Br_2_ (0.45 mg, 0.0025 mmol, 0.05 eq.), and DMSO (2 mL) were added to a 20 mL vial containing the stir bar wrapped with copper wire. The vial was sealed with a septum lid. To increase the solubility of the ATRP initiator, the mixture was warmed up to 35°C and sparged with N_2_ for 30 min. To start the reaction, PMDETA (0.002 mL, 0.001 mmol, 0.3 eq.) was added, and the mixture was stirred at room temperature for 2 h. The viscous mixture was diluted with acetone before being passed through a silica column to remove the copper. Excess acetone was removed in vacuo, and the product was purified twice by precipitation against cold diethyl ether. The precipitate was dried under vacuum at 60 °C to yield 0.98 g (49%) **pOEGMA-NDI** as a colorless viscous liquid.

^1^H-NMR (400 MHz, DMSO) 4.20–3.30 (m, OCH_2_CH_2_O), 3.44 (s, OCH_3_), 2.5 (m, backbone/aliphatic protons). GPC (DMF): *M*_n_ = 32 kg mol^-1^; *Đ* =2.3

**Scheme S5.** Synthesis of **pOEGMA-BPEA.**

**Synthesis of** **pOEGMA-BPEA.** Compound **D1** (150 mg, 0.34 mmol, 1 eq.) was dissolved in dry THF (50 mL) in a 100 mL flask equipped with a stir bar, and triethylamine (0.2 mL, 1.4 mmol, 2.4 eq.) was added. The solution was cooled to 0 °C in an ice bath, and $\alpha$-bromoisobutyryl bromide (188 mg, 0.82 mmol, 2.4 eq.) was added dropwise over the course of 20–30 min. The reaction mixture was stirred at 0 °C for 30 min, and then stirred at room temperature for 18h. The formed by-product precipitate was filtered off, the solvent was removed under reduced pressure, and the product was purified by flash column chromatography on silica gel (gradient to 50/50 v/v% hexanes/ethyl acetate) to afford **D3** (120 mg, 48%) as a bright yellow solid.

^1^H-NMR (400 MHz, CHCl_3_) δ: 8.61 (q, 4H, ArH), 7.71 (d, J = 7.9 Hz, 2H, ArH), 7.60 (q, J = 7.9 Hz, 2H, ArH), 7.43 (d, J = 7.9 Hz, 4H, ArH), 5.22 (s, 2H, CH_2_O), 1.92 (s, 12H, CH_3_). Exact Mass: 589.11, found mass: 587.12 ([M+H]^+^)

OEGMA was passed through a column of basic alumina. A copper wire (~10 cm) was sanded to remove the oxide surface layer, washed with acetone, and wrapped around a cylindrical stir bar OEGMA_500_ (2.00g, 4.00 mmol, 100 eq.)compound **D3** (30 mg, 0.04 mmol, 1 eq.), Cu(II)Br_2_ (0.45 mg, 0.0025 mmol, 0.05 eq.), and DMSO (2 mL) were added to a 20 mL vial containing the stir bar wrapped with copper wire. The vial was sealed with a septum lid. To increase the solubility of the ATRP initiator, the mixture was warmed up to 35°C and sparged with N_2_ for 30 min. To start the reaction, PMDETA (0.002 mL, 0.001 mmol, 0.3 eq.) was added, and the mixture was stirred at room temperature for 2 h. The viscous mixture was diluted with acetone before being passed through a silica column to remove the copper. Excess acetone was removed in vacuo, and the product was purified twice by precipitation against cold diethyl ether. The precipitate was dried under vacuum at 60 °C to yield 1.00 g (49%) **pOEGMA-BPEA** as yellow-green viscous liquid.

^1^H-NMR (400 MHz, DMSO) 4.20–3.30 (m, OCH_2_CH_2_O), 3.44 (s, OCH_3_), 2.5 (m, backbone/aliphatic protons). GPC (DMF): *M*_n_ = 43 kg mol^-1^; *Đ* =2.5.

**Preparation of pAAm Hydrogels**

Hydrogels were prepared by redox-initiated free-radical polymerization of acrylamide in aqueous solution at room temperature, without prior degassing or the use of a nitrogen atmosphere.

**Preparation of Stock Solutions.** Prior to hydrogel preparation, stock solutions of acrylamide (AAm, 4.00 g in 10 mL deionized water), N,N′-methylenebis(acrylamide) (MBA, 50 mg in 5 mL deionized water), ammonium persulfate (APS, 0.45 g in 1 mL deionized water), and tetramethylethylenediamine (TEMED, 0.3 mL in 0.7 mL deionized water) were prepared. Stock solutions of **pOEGMA-NDI**, **pOEGMA-BPEA**, **pOEGMA-NDI-BPEA**, and **pOEGMA-Loop** were prepared by dissolving 0.6-0.7 mg of each polymer in 1 mL of deionized water. All stock solutions were stored at 4 °C before use.

**General Procedure for Hydrogel Preparation.** For the preparation of three hydrogel samples (three disks or three strips), ~1 mL of precursor solution was prepared in a 1.5 mL glass vial. Thus, 0.50 mL of the corresponding pOEGMA stock solution (or 0.5 mL deionized water for the neat pAAm reference hydrogel), 0.50 mL of AAm stock solution, and 50 μL of MBA stock solution were combined and mixed thoroughly by vortexing. Subsequently, 3.0 μL of APS stock solution and 3.0 μL of TEMED stock solution were added, and the mixture was briefly vortexed in the closed vial. Polymerization was initiated within a few minutes after the addition of APS and TEMED. The required volume of precursor solution was immediately transferred into the desired mold (**Table S1**), and the gels were allowed to polymerize and set in the covered mold at room temperature for 30 min.

Using the general procedure described above, the following hydrogel formulations were prepared:

(0) **Neat pAAm** hydrogel: precursor solution prepared without pOEGMA additive.
(1) **pAAm-NDI** hydrogel: precursor solution prepared using pOEGMA-NDI stock solution.
(2) **pAAm-BPEA** hydrogel: precursor solution prepared using pOEGMA-BPEA stock solution.
(3) **pAAm-NDI-BPEA** hydrogel: precursor solution prepared using pOEGMA-NDI-BPEA stock solution.
(4) **pAAm-Loop** hydrogel: precursor solution prepared using pOEGMA-Loop stock solution.

**Preparation of pOEGMA Hydrogels**

Hydrogels were prepared by redox-initiated free-radical polymerization in aqueous solution at room temperature, without prior degassing or the use of a nitrogen atmosphere.

**Preparation of Stock Solutions.** Prior to hydrogel preparation, the following stock solutions were prepared in deionized water: oligo(ethylene glycol) methyl ether methacrylate (OEGMA₅₀₀, 1.00 g in 1 mL), poly(ethylene glycol) dimethacrylate (PEGDMA₇₅₀, 0.5 g in 1 mL), ammonium persulfate (APS, 0.45 g in 1 mL deionized water), and tetramethylethylenediamine (TEMED, 0.3 mL in 0.7 mL deionized water). A **pOEGMA-Loop** stock solution was prepared by dissolving 0.6 mg of **pOEGMA-Loop** in 1 mL of deionized water. All stock solutions were stored at 4 °C before use. It should be noted that commercial OEGMA₅₀₀ may contain trace amounts of dimethacrylate impurities, which can vary between batches. Consequently, slight variations in the cross-linking density and mechanical properties of the resulting hydrogels may be observed.

**General Procedure for Hydrogel Preparation.** For each hydrogel formulation, ~1 mL of precursor solution was prepared by combining 0.50 mL of OEGMA₅₀₀ stock solution, 0.50 mL of the **pOEGMA-Loop** stock solution, and 10 μL of PEGDMA₇₅₀ stock solution in a 1.5 mL vial and mixed thoroughly by vortexing. Polymerization was initiated by the sequential addition of 3.0 μL of APS stock solution and 3.0 μL of TEMED stock solution, followed by brief vortexing. The precursor solution was immediately transferred into the desired mold (**Table S1**), and the gels were allowed to polymerize and set at room temperature for 30 min.

**Table S1**. Sample geometries and precursor volumes used for mechanical and swelling experiments.

| **Experiment** | **Gel system** | **Mold dimensions^a^** | **Precursor volume** |
| --- | --- | --- | --- |
| **Stretching** | pAAm or OEGMA | 5 mm × 25 mm  rectangular strip | 0.3 mL |
| **Swelling / compression** | pAAm or OEGMA | 8 mm diameter  circular disk | 0.3mL |

^a^ The mold for rectangular strips was fabricated in-house from PTFE and contained three cavities (5 mm × 25 mm). The molds were covered with a glass slide during gelation. Disk-shaped samples were prepared using the barrel of a 2 mL syringe as a mold after removal of the tip and were closed with a conical joint stopper (BRAND®).

**Probing the Inertness of pOEGMA-Loop during the Polymerization of pAAm**

For the polymerization of neat pAAm, acrylamide (0.40 g in 1 mL in deionized water), APS (2.0 μL 2 M aqueous solution), and TEMED 2.0 μL 2M aqueous solution) were vortexed, without further degassing in a 4 mL vial, and the mixture was allowed to polymerize for 30 min at room temperature. The viscous mixture was further diluted with deionized water and prepared for the SEC measurements in DMF. In a 1.5 mL vial, acrylamide (0.40 g in 1 mL in deionized water), **pOEGMA-Loop** (1.00 mg), APS (2.0 μL 2 M aqueous solution), and TEMED 2.0 μL 2M aqueous solution) were vortexed, and the mixture was allowed to polymerize for 30 min at room temperature. The viscous reaction mixture was then precipitated into THF (5 mL), affording a white precipitate and a yellow solution phase. The white precipitate was isolated by filtration, redissolved in water, and retained as the THF-insoluble fraction. The yellow THF phase was collected, and the solvent was removed to afford the THF-soluble fraction (0.65 mg). (**Figure S3**). Both fractions were analyzed by SEC with dRI and UV detection to evaluate the distribution of **pOEGMA-Loop** between the soluble and insoluble polymer fractions. The THF-soluble fraction displays SEC traces similar to those of pOEGMA-Loop, with no significant change in molecular weight (*M*_n_ = 32 kg mol^−1^, *Đ* = 2.6). The THF-insoluble fraction shows traces resembling the neat pAAm reference (**Figure S2c**). While the presence of **pOEGMA-Loop** in the insoluble fraction cannot be ruled out because this region overlaps with the pAAm signal, the recovery of most of the pOEGMA mass (65%) in the THF-soluble fraction reflects that the majority of the additive does not experience any chain transfer during polymerization. UV–vis absorbance analysis of the THF-soluble fraction confirmed the presence of **pOEGMA-Loop** (**Figure S3**), whereas the THF-insoluble fraction exhibits only minimal absorbance features associated with **pOEGMA-Loop**.

**Swelling Experiments**

Hydrogel disks (d = 8 mm) were weighed in the as-prepared state ($m_{0}$ = 0.26 ± 0.012 g). The fluorescence emission spectra were then measured using the *in situ* PL set-up described above in the General Methods section (Page S2)*.* The gels were subsequently immersed in deionized water in covered Petri dishes and allowed to swell to equilibrium overnight at room temperature. After equilibration, excess surface water was carefully removed with a lint-free tissue, and the swollen gels were weighed again ($m_{\mathrm{eq}}$ = 2.09 ± 0.090 g), indicating a relative mass increase *S* = 695 ± 9%, independent of the composition. To restore the hydrogels to their as-prepared state after swelling, the swollen samples were placed on a non-absorbent, perforated aluminum dish and covered with perforated aluminum foil to minimize rapid water evaporation while allowing uniform drying. The samples were dried at room temperature for 24-36h, and their mass was monitored periodically throughout the drying process. Optical characterization of the dried hydrogels was performed once the samples had reached approximately the same mass (m ~ 2.5 g) and dimensions (d ~ 8 mm) as the as-prepared hydrogels. PL spectra were then recorded under identical instrumental conditions for the as-prepared, swollen, and dried hydrogel samples. The relative mass increase upon swelling *S* (%) was determined from the initial ($m_{0}$) and equilibrium swollen ($m_{\mathrm{eq}}$) weights of the corresponding hydrogel samples by S = (*m_eq_*-*m*_0_)/m_0_.

For UV–Vis absorption measurements, hydrogel strips were used. Immediately after preparation, each gel strip was placed in a PMMA cuvette, and its absorption spectrum was recorded. The cuvette was then filled with deionized water, and the gel was allowed to swell to equilibrium overnight at room temperature. After swelling, the water was removed from the cuvette, and the absorption spectrum of the swollen hydrogel was recorded.

**Tensile Deformation Experiments**

Hydrogel strips (17.2 x 5 x 2 mm) were mounted between the clamps of a Linkam TST350 microtensile tester and subjected to uniaxial tensile deformation at room temperature. PL spectra were recorded *in* *situ* throughout the deformation by positioning a fiber-optic probe at an angle of 45° relative to the sample surface as described above in the General Methods section (Page S2)*.* The emission intensity was monitored as a function of applied strain. For cyclic loading experiments, repeated stretch–release cycles were performed between 0 and 430% strain, while stress-relaxation experiments were carried out by stretching the samples to 430% strain and maintaining the strain constant while monitoring the fluorescence intensity over time. The tensile strain was calculated as:

$$\varepsilon_{\mathrm{tensile}}(\%)=\frac{L-L_{0}}{L_{0}}\times100$$

where $L_{0}$is the initial gauge length and $L$ is the gauge length under deformation.

**Compression Experiments**

Hydrogel disks ($h_{0}$ = 2.5 ± 0.12 mm) were placed between two glass slides and subjected to cyclic compressive deformation ($h$= 0.8 ± 0.05 mm). Fluorescence emission spectra were recorded *in situ* by positioning a fiber-optic probe at an angle of 45° relative to the sample surface. The emission intensity was monitored as a function of compressive strain over repeated compression–release cycles. The compressive strain was calculated as:

$$\varepsilon_{\mathrm{compression}}(\%)=\frac{h_{0}-h}{h_{0}}\times100$$

where $h_{0}$is the initial sample thickness and $h$ is the thickness during compression.

**Supplementary Figures**

**Figure S1.** SEC traces, recorded in DMF, of the various pOEGMA-based additives. a) **pOEGMA-NDI**, b) **pOEGMA-BPEA**, and c) **pOEGMA-Loop**. The elugrams shown were recorded by refractive index (dRI, black) and UV (dUV, purple) detectors.

**Figure S2.** SEC traces, recorded in DMF, collected in a control experiment probing the inertness of **pOEGMA-Loop** during the polymerization of pAAm (Page S10). a) SEC traces of the THF-soluble fraction, which contains primarily the **pOEGMA-Loop** additive, recorded by dRI (black) and dUV (purple) detectors. The SEC traces are virtually identical to those of the pristine additive (**Figure S1a**), demonstrating the absence of any covalent bond formation through reaction with the propagating pAAm chain radicals. b) THF-insoluble fraction, which contains mainly pAAm formed during the reaction. c) The SEC traces, recorded in DMF, of a neat linear pAAm reference monitored by dRI (black) and dUV (purple) detectors show similar profiles.

**Figure S3.** a) Pictures taken during a control experiment probing the inertness of **pOEGMA-Loop** during the polymerization of pAAm (Page S10). The images show 1) The reaction mixture containing acrylamide (AAm), **pOEGMA-Loop**, APS, and TEMED, 10 min after initiation, showing pronounced viscosity increase; 2) An inverted vial after precipitation of the polymerization mixture into THF, showing a white precipitate (pAAm) and a yellow THF-soluble fraction (**pOEGMA-Loop**); 3) A vial containing the pAAm precipitate redissolved in water; 4) A vial containing the THF-soluble phase. b) Absorbance spectra of the THF-insoluble phase (red) and the THF-soluble phase (black), confirming that the **pOEGMA-Loop** resides primarily or fully in the THF phase.

**Figure S4. a)** Non-normalized PL spectra of **A1**, **D1**, an equimolar mixture of **A1** and **D1**, and the **D-A Loop** in THF at 10 μM. b) PL spectra of the mechanochromic additives **pOEGMA-Loop, pOEGMA-BPEA**, and an equimolar mixture of **pOEGMA-NDI** and **pOEGMA-BPEA** in water. The additive concentrations correspond to dye concentrations of c ~ 10 μM.  All measurements were performed with an excitation wavelength λ_ex_ = 410 nm.

**Figure S5.** Concentration dependence of the quenching effect in equimolar mixtures of NDI (**A1**) and BPEA (**D1**) model compounds, and the **D-A Loop**. Shown are representative PL spectra of **D1**, an equimolar mixture of **A1** and **D1**, and the **D-A Loop** in THF at different concentrations between 0.6 and 5 μM. a) Normalized spectra relative to the maximum intensity of **D1** at 476 nm, which was set to 100%. b) Non-normalized spectra. All measurements were performed with an excitation wavelength *λ*_ex_ = 410 nm.

**Figure S6.** Concentration-dependence of the quenching effect in equimolar mixtures of NDI (**A1**) and BPEA (**D1**) model compounds, and the **D-A Loop**. Shown is the residual BPEA fluorescence as a function of concentration of the equimolar **A1** & **D1** mixture and the **D–A Loop** in THF. The residual fluorescence was calculated from the spectra shown in **Figure S5a** as the emission intensity relative to the BPEA model compound (**D1**) at the same concentration (I/I_D1_× 100%). Data points shown are averages of three samples, with error bars indicating the standard deviation**.**

**Figure S7.** PL spectra of the as-prepared (solid line) and 700% water-swollen (dashed line) **pAAm-NDI** hydrogel. The spectra were collected at λ_ex_ = 405 nm.

**Figure S8.** Absorbance spectra of a) **pAAm-NDI** b) **pAAm-BPEA** c) **pAAm-NDI-BPEA**, and d) **pAAm-Loop** hydrogels in the as-prepared (solid line) and water-swollen (700%) state (dashed line). Minor differences in absorbance intensity are attributed to small variations in hydrogel thickness between samples.

**Figure S9.** Pictures of **pAAm-NDI** (1), **pAAm-BPEA** (2), **pAAm-NDI-BPEA** (3), and **pAAm-Loop** (4) hydrogels, as prepared (top) and after swelling in water to 700% of their original weight (bottom) under ambient light.

**Figure S10.** Stress–strain curve recorded during stretching the neat pAAm hydrogel reference.

**Figure S11.** a) PL spectra of the **pAAm-Loop** hydrogel in the force-free and strained (430% strain) state recorded over 12 stretching–relaxation cycles. b) PL spectra of the **pAAm-Loop** hydrogel in the compressed (ε = 68% compressive strain) and relaxed states, recorded over 5 compression–relaxation cycles. c) Mechanoresponse of a **pAAm-Loop** gel upon cyclic compression. The plot shows the emission intensity of the gel in force-free and compressed (68%) states, recorded over 12 stretching-relaxation cycles. All spectra were collected with an excitation wavelength, λ_ex_, of 405 nm.

**Figure S12.** a) Stress relaxation experiment. The plot shows the PL emission spectra recorded after stretching a **pAAm-Loop** gel to a strain of ε = 380% and fixing the strain. The spectra were recorded at intervals of 120 sec over 1600 sec with an excitation wavelength λ_ex_ = 405 nm.

**Figure S13.** Comparison of the mechanochromic responses of **pOEGMA-Loop** hydrogels in response to stretching, swelling and compression. a) PL spectra recorded during gradual stretching of a **pOEGMA-Loop** hydrogel. b) PL spectra of a **pOEGMA-Loop** hydrogel in the as-prepared (solid line) and swollen (dashed line) state. c**)** PL spectra of **pOEGMA-Loop** hydrogel in the compressed (60 % compression) and relaxed state. The spectra were collected with an excitation wavelength, λ_ex_, of 405 nm

**Calculations**

**Estimation of the fraction of activated Loop mechanophores in the pAAm-Loop hydrogels**

To quantify the fraction of Loop mechanophores in the **pAAm-Loop** hydrogels that are mechanically activated upon deformation (or are active in the idle state), the fluorescence intensity of the **pAAm-Loop** hydrogel was compared to that of the control **pAAm-BPEA** hydrogel. The control hydrogel was measured using the same protocol and serves to compensate for purely geometric effects, such as sample thinning and changes in optical path length.

For each strain ε, the emission intensities at 478 nm of the **pAAm-Loop** and **pAAm-BPEA** hydrogels, $I_{Loop}\left( \varepsilon\right)$ and $I_{Loop}\left( \varepsilon\right)$ were recorded. A geometry-corrected intensity was obtained by scaling the measured **pAAm-Loop** intensity with the ratio of the undeformed and strained intensities of the **pAAm-BPEA** control:

$$I_{Loop, corr} =I_{Loop}\left( \varepsilon\right)\frac{I_{BPEA}\left( 0 \right)}{I_{BPEA}\left( \varepsilon\right)}$$

where:

$I_{Loop, corr}$= emission intensity of the **pAAm-Loop** hydrogel,

$I_{\mathrm{Loop}}(\varepsilon)$ = emission intensity of the **pAAm-Loop** hydrogel at strain $\varepsilon$,

$I_{\mathrm{BPEA}}(0)$ = emission intensity of the **pAAm-BPEA** hydrogel at the same strain 0,

$I_{\mathrm{BPEA}}(\varepsilon)$ = emission intensity of the **pAAm-BPEA** hydrogel at the same strain $\varepsilon$.

**Table S2.** Emission intensities at 478 nm for **pAAm-BPEA**, **pAAm-Loop** and geometry‑corrected $I_{\text{Loop,corr}}$for the **pAAm‑Loop** hydrogel.

| **Strain [%]** | $\boldsymbol{I}_{\boldsymbol{BPEA}}$**[CPS]** | $\boldsymbol{I}_{\boldsymbol{Loop}}$ **[CPS]** | $\boldsymbol{I}_{\boldsymbol{Loop, corr}}$ **[CPS]** |
| --- | --- | --- | --- |
| 0 | 277.41 | 22.72 | 22.72 |
| 72 | 201.44 | 32.55 | 44.83 |
| 143 | 175.09 | 43.44 | 68.83 |
| 215 | 153.38 | 63.24 | 114.38 |
| 286 | 143.74 | 72.49 | 139.90 |
| 358 | 134.35 | 98.54 | 203.47 |
| 430 | 122 | 118.54 | 269.54 |

The fraction of activated Loop mechanophores, $f_{ON}$ , was then estimated by normalizing the corrected Loop intensity between the undeformed **pAAm-Loop** hydrogel (quenched) and the undeformed **pAAm-BPEA** hydrogel (fully unquenched reference, 100% activation):

$$f_{ON}=\frac{I_{Loop, corr}\left( \varepsilon\right)-I_{Loop}(0)}{I_{BPEA}\left( \varepsilon\right)-I_{Loop}(0)}$$

where,

$I_{\mathrm{Loop}}(\varepsilon)$ = emission intensity of the **pAAm-Loop** hydrogel at strain $\varepsilon$,

$I_{\mathrm{BPEA}}(\varepsilon)$ = emission intensity of the **pAAm-BPEA** hydrogel at the same strain $\varepsilon$,

$I_{\mathrm{Loop}}(0)$ = emission intensity of the **pAAm-Loop** hydrogel at zero strain.

**Table S3.** Estimation of the fraction of activated Loop mechanophores in the pAAm-Loop hydrogels as a function of strain. The values were calculated from the emission spectra shown in **Figure 4** of the main manuscript.

| **Strain [%]** | $\boldsymbol{f}_{\boldsymbol{ON}}$ |
| --- | --- |
| 0 | 0.00 |
| 72 | 0.09 |
| 143 | 0.18 |
| 215 | 0.36 |
| 286 | 0.46 |
| 358 | 0.71 |
| 430 | 0.97 |

**Estimation of the molecular weight between cross-links (*M*_c_) in pAAm**

The theoretical average molar mass of polymer strands between cross-linking points, *M*_c_ , was estimated from the monomer and cross-linker feed composition according to Wisniewska et al.:^[1]^

$$d_{c}=\frac{n_{MBA}}{n_{AAm}+n_{MBA}}$$

and

$$M_{c}={\frac{1}{2}M}_{0}d_{c}^{-1}$$

where $d_{c} \mathrm{is}$the degree of cross-linking, and $n_{MBA}$ and $n_{AAm}$ are the molar amounts of methylenebis(acrylamide) (MBA) and acrylamide (AAm), respectively, and $M_{0}$ is the molar mass of the AAm repeat unit (71.08 g mol ^-1^). For the near pAAm, $n_{AAm}$ = 2.81 mmol and $n_{MBA}$ = 3.24 μmol, affording an $M_{c}$ = 32 kg mol^-1^.**NMR Data**

**Figure S14.** ^1^H NMR spectrum (DMSO_3_, 400 MHz) of **A1**.

**Figure S15.** ^1^H NMR spectrum (CDCl_3_, 400 MHz) of **A2**.

**Figure S16.** ^1^H NMR spectrum (CDCl_3_, 400 MHz) of **D1**.

**Figure S17.** ^1^H NMR spectrum (CDCl_3_, 400 MHz) of **D2**.

**Figure S18.** ^1^H NMR spectrum (CDCl_3_, 400 MHz) of **D-A Loop**.

**Figure S19.** ^1^H NMR spectrum (DMSO, 400 MHz) of **pOEGMA-Loop**.

**Figure S20.** ^1^H NMR spectrum (CDCl_3_, 400 MHz) of **A3**.

**Figure S21.** ^1^H NMR spectrum (CDCl_3_, 400 MHz) of **A4**.

**Figure S22.** ^1^H NMR spectrum (DMSO, 400 MHz) of **pOEGMA-NDI**.

**Figure S23.** ^1^H NMR spectrum (CDCl_3_, 400 MHz) of **D3.**

**Figure S24.** ^1^H NMR spectrum (CDCl_3_, 400 MHz) of **pOEGMA-BPEA**.

**Supporting Reference**

[1]	M. A. Wisniewska, J. G. Seland, W. Wang, “Determining the scaling of gel mesh size with changing crosslinker concentration using dynamic swelling, rheometry, and PGSE NMR spectroscopy” *J. Appl. Polym. Sci* **2018**, *135*, 46695.
